# RBOHF activates stomatal immunity by modulating both reactive oxygen species and apoplastic pH dynamics in Arabidopsis

**DOI:** 10.1111/tpj.16380

**Published:** 2023-07-14

**Authors:** Dominique Arnaud, Michael J. Deeks, Nicholas Smirnoff

**Affiliations:** ^1^ Biosciences, Faculty of Health and Life Sciences University of Exeter Exeter EX4 4QD UK

**Keywords:** *Arabidopsis thaliana*, stomatal immunity, reactive oxygen species, *Pseudomonas syringae*, redox biosensor

## Abstract

Stomatal defences are important for plants to prevent pathogen entry and further colonisation of leaves. Apoplastic reactive oxygen species (ROS) generated by NADPH oxidases and apoplastic peroxidases play an important role in activating stomatal closure upon perception of bacteria. However, downstream events, particularly the factors influencing cytosolic hydrogen peroxide (H_2_O_2_) signatures in guard cells are poorly understood. We used the H_2_O_2_ sensor roGFP2‐Orp1 and a ROS‐specific fluorescein probe to study intracellular oxidative events during stomatal immune response using Arabidopsis mutants involved in the apoplastic ROS burst. Surprisingly, the NADPH oxidase mutant *rbohF* showed over‐oxidation of roGFP2‐Orp1 by a pathogen‐associated molecular pattern (PAMP) in guard cells. However, stomatal closure was not tightly correlated with high roGFP2‐Orp1 oxidation. In contrast, RBOHF was necessary for PAMP‐mediated ROS production measured by a fluorescein‐based probe in guard cells. Unlike previous reports, the *rbohF* mutant, but not *rbohD*, was impaired in PAMP‐triggered stomatal closure resulting in defects in stomatal defences against bacteria. Interestingly, RBOHF also participated in PAMP‐induced apoplastic alkalinisation. The *rbohF* mutants were also partly impaired in H_2_O_2_‐mediated stomatal closure at 100 μm while higher H_2_O_2_ concentration up to 1 mm did not promote stomatal closure in wild‐type plants. Our results provide novel insights on the interplay between apoplastic and cytosolic ROS dynamics and highlight the importance of RBOHF in plant immunity.

## Significance Statement

Whilst most studies have focused on the NADPH oxidase RBOHD, the role of RBOHF in plant defence responses to pathogens in Arabidopsis has been largely underestimated for decades. We show that RBOHF promotes stomatal immunity and Arabidopsis resistance to *Pseudomonas syringae* bacteria by affecting not only ROS signalling but also by modulating apoplastic pH, two processes probably interconnected via RBOHF during the response to pathogen‐associated molecular patterns.

## INTRODUCTION

Plants are continuously exposed to diverse microorganisms and must develop strategies to avoid infection by restricting the entry and multiplication of pathogens. The main first line of plant defences against foliar bacteria is the closure of stomatal pores at the leaf epidermis. Stomata are formed by a pair of guard cells that mediate gas exchange between the plant and the environment and are critical during the plant's innate immune response (Melotto et al., 2006; Melotto et al., 2008). Stomatal immunity is initiated by the recognition of pathogen‐associated molecular patterns (PAMPs) by pattern recognition receptors at the plasma membrane. In *Arabidopsis thaliana*, the receptor FLAGELLIN‐SENSITIVE2 (FLS2) that recognises the peptide flg22, the biologically active epitope of the bacterial PAMP flagellin, plays a prominent role in stomatal immunity (Melotto et al., 2006; Zeng & He, 2010). Downstream of PAMP perception, calcium, reactive oxygen species (ROS), nitric oxide and apoplastic alkalinisation function as secondary messengers to promote stomatal closure (Arnaud & Hwang, 2015). Stomatal immunity was also shown to be modulated by hormonal pathways, mainly the abscisic acid (ABA) and salicylic acid (SA) signalling pathways (Arnaud & Hwang, 2015; Melotto et al., 2008). To counteract PAMP‐mediated stomatal closure, pathogens have evolved virulence factors, such as the phytotoxin coronatine (COR) secreted by the bacterial pathogen *Pseudomonas syringae* pv tomato (*Pst*) strain DC3000, to counteract host stomatal defences by promoting stomatal reopening (Melotto et al., 2006).

During the immune response, the apoplastic ROS burst is mainly generated by the NADPH oxidase isoform RBOHD and to a lesser extent RBOHF (Torres et al., 2002; Zhang et al., 2007). While RBOHD participates in stomatal (pre‐invasive) defence responses, its contribution to plant resistance against *Pst* bacteria is not clear (Chaouch et al., 2012; Kadota et al., 2014; Zhang et al., 2007). Moreover, the role of RBOHD in PAMP‐triggered stomatal closure has been questioned (Guzel Deger et al., 2015; Khokon et al., 2010). On the contrary, RBOHF is required for full post‐invasive (late apoplastic) resistance against virulent *Pst* bacteria (Chaouch et al., 2012) and no role in stomatal defence has been described until now. The type III cell wall peroxidases PRX33 and PRX34 also participate in PAMP‐mediated ROS production and play an important role in both pre‐ and post‐invasive defences against *Pst* bacteria (Arnaud et al., 2017; Daudi et al., 2012). Although it is unknown how PAMPs activate PRXs, apoplastic alkalinisation is a prerequisite for PRX‐mediated apoplastic ROS production (Bolwell et al., 2002). Apoplastic ROS, mainly hydrogen peroxide (H_2_O_2_), diffuse into the cytosol through plasma membrane aquaporins (Rodrigues et al., 2017; Tian et al., 2016). However, recent results indicate that PAMP‐ and bacteria‐induced oxidation of the cytosolic/nuclear localised H_2_O_2_ sensor roGFP2‐Orp1 in leaf discs is largely independent of RBOHD, RBOHF, PRX33 and PRX34 (Arnaud et al., 2023). Moreover, it remains unclear how apoplastic ROS produced by NADPH oxidases or cell wall peroxidases upon PAMP perception influences intracellular H_2_O_2_/ROS accumulation specifically in guard cells.

In this contribution, we used three different probes to analyse oxidative events after flg22 treatment. The chemiluminescent luminol and fluorescein H_2_DCFDA probes detect, respectively, extracellular and intracellular ROS and are not specific to H_2_O_2_ (Murphy et al., 2022). In contrast, the ratiometric biosensor roGFP2‐Orp1 localised in the cytosol and nucleus is mainly oxidised by H_2_O_2_ *in vivo* and can be reduced by the glutaredoxin/glutathione system permitting dynamic and real‐time measurements (Arnaud et al., 2023; Nietzel et al., 2019). We show that PAMP‐triggered oxidation of roGFP2‐Orp1 is surprisingly stronger in guard cells of the *rbohF*, *prx33‐3* and *prx34‐2* mutants compared to WT plants. Despite an increase in H_2_O_2_ in guard cells upon PAMP perception, we found a weak negative correlation between stomatal aperture and roGFP2‐Orp1 oxidation. Moreover, roGFP2‐Orp1 oxidation state in guard cells was not correlated with a stomatal aperture in control conditions. More importantly, we found that RBOHF plays a crucial role in stomatal immunity and defence against *Pseudomonas syringae* bacteria by a complex control of both ROS production and apoplastic pH.

## RESULTS

### PAMP‐induced oxidation of the H_2_O_2_ sensor roGFP2‐Orp1 is higher in guard cells of the *rbohF* mutant

**Figure 1:**
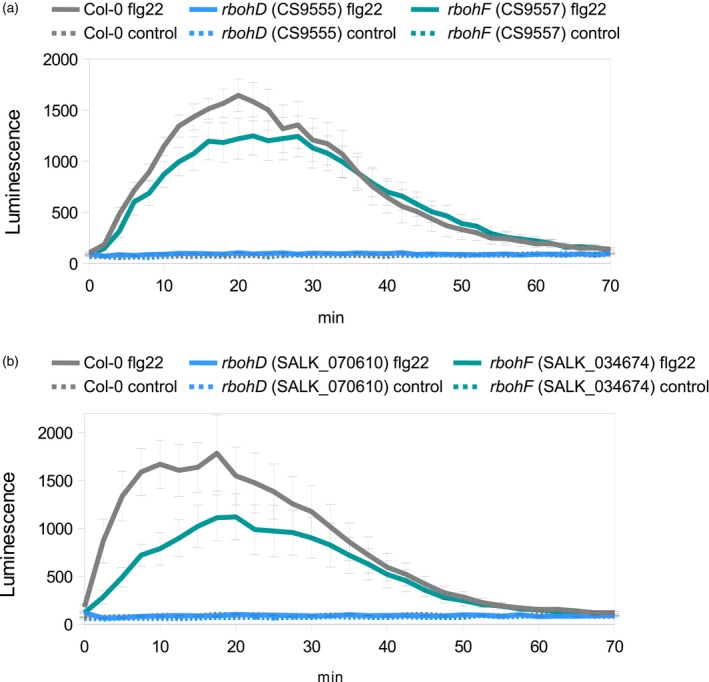
The *rbohD* mutants are defectives in PAMP‐triggered ROS burst in the apoplast. (a and b) PAMP‐induced apoplastic ROS production detected by luminol assay in Col‐0 WT, *rbohD* (CS9555) and *rbohF* (CS9557) mutants (a) and other allelic *rbohD* (SALK_070610) and *rbohF* (SALK_034674) mutants (b). The luminescence was measured over time after treatment with control solution or 1 μm flg22 at *t* = 0 min. Data are means ± SE (*n* = 6) from a representative experiment.

**Figure 2:**
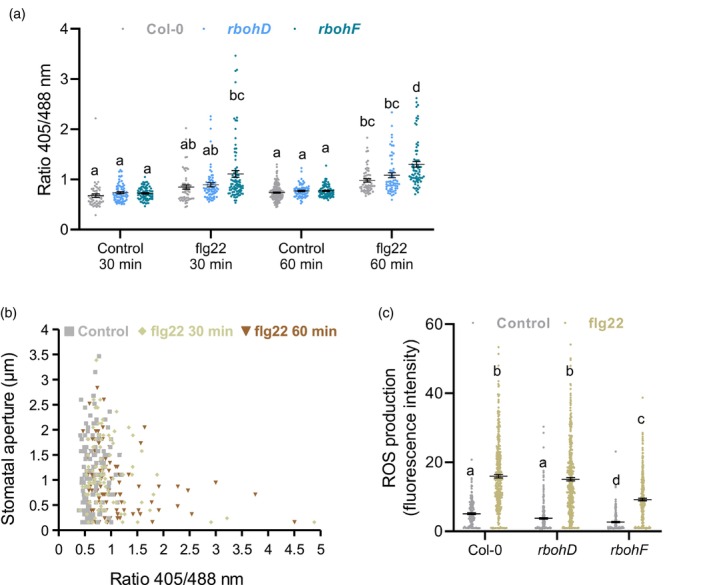
The H_2_O_2_ sensor roGFP2‐Orp1 is more oxidised in guard cells of the *rbohF* mutant. (a) Oxidation state of roGFP2‐Orp1 in Col‐0 WT, *rbohD* and *rbohF* guard cells in response flg22. Leaf discs were exposed to control solution or 1 μm flg22 and the ratio 405/488 nm of stomata was quantified at 30 min and 60 min after treatment from images of the fluorescence emission at 517 ± 9 nm following excitation at 488 and 405 nm. Data are means ± SE (*n* ≥ 50 guard cells) from a representative experiment. Different letters indicate significant differences at *P* < 0.05 based on a Tukey's HSD test. (b) Correlation between the stomatal aperture and the oxidation state of roGFP2‐Orp1 in guard cells of leaf discs after treatment with control solution or 1 μm flg22 for 30 min and 60 min. The scatter plots show the stomatal aperture as a function of the ratio 405/488 nm for each condition (*n* ≥ 100). The Pearson correlation coefficient (R) is 0.0651 (*P* = 0.483) for control treatment and − 0.2428 (*P* = 0.015) and − 0.2395 (*P* = 0.011) for flg22 treatment at 30 min and 60 min, respectively. (c) ROS production detected by H_2_DCFDA fluorescence in guard cells of Col‐0 WT, *rbohD* and *rbohF* epidermal peels 30 min after treatment with control solution or 5 μm flg22. Data are means ± SE (*n* ≥ 200) from 3 independent experiments. Different letters indicate significant differences at *P* < 0.05 based on a Tukey's HSD test.

Using the chemiluminescent luminol probe to detect extracellular ROS production, RBOHD was shown to be the main NADPH oxidase implicated in PAMP‐induced ROS burst in the apoplast of Arabidopsis leaves (Mersmann et al., 2010; Zhang et al., 2007). Indeed, luminol assays performed on leaf discs showed that the *rbohD* mutants were defective in flg22‐triggered apoplastic ROS burst while a non‐significant decrease in ROS production was observed in the *rbohF* mutants compared to Col‐0 WT (Figure 1). Surprisingly, while flg22 causes stomatal closure, ROS production in the cytosol of *rbohD* or *rbohF* guard cells upon flg22 activation has not been reported. Moreover, these two NADPH oxidases were not implicated in intracellular ROS increase in guard cells after treatment with yeast elicitors (Khokon et al., 2010). To analyse the contribution of apoplastic ROS burst to cytosolic H_2_O_2_ accumulation during the immune response, we used Arabidopsis lines in which the H_2_O_2_ sensor roGFP2‐Orp1 has been introduced into the *rbohD*, *rbohF*, *prx33‐3* and *prx34‐2* mutants, respectively (Arnaud et al., 2023). We analysed PAMP‐triggered roGFP2‐Orp1 oxidation in pavement cells and guard cells of leaf discs after dual excitation at 405 nm and 488 nm (emission at 508–526 nm) by confocal laser scanning microscopy (Figure 2, Figure S1). No significant difference in roGFP2‐Orp1 oxidation state was observed in *rbohD*, *rbohF*, *prx33‐3* and *prx34‐2* pavement cells and guard cells as compared to Col‐0 in control conditions (Figure S1a,b). The *rbohD*, *prx33‐3* and *prx34‐2* mutants were not affected in roGFP2‐Orp1 oxidation in pavement cells at 60 min after flg22 treatment. Surprisingly, the *rbohF* mutant exhibited a marked increase in flg22‐mediated roGFP2‐Orp1 oxidation compared to Col‐0 in pavement cells (Figure S1a) suggesting lower antioxidant capacities in this mutant as observed before (Arnaud et al., 2023).

In Col‐0 guard cells, roGFP2‐Orp1 oxidation started to increase from 20 min after flg22 treatment (Figure S1d). The *rbohD* mutant was not impaired in flg22‐mediated roGFP2‐Orp1 oxidation in guard cells (Figure 2a). By contrast, the *rbohF* mutant showed higher roGFP2‐Orp1 oxidation compared to Col‐0 WT at 30 and 60 min after flg22 treatment. Similarly, the *prx33‐3* and *prx34‐2* mutants which are impaired in stomatal immunity (Arnaud et al., 2017) showed a significant increase in flg22‐induced roGFP2‐Orp1 oxidation in guard cells as compared to Col‐0 (Figure S1b). These unexpected results suggest that a defect in PAMP‐mediated stomatal closure is correlated with an enhanced H_2_O_2_ or oxidation state of guard cell cytosol/nucleus. A more detailed comparison of roGFP2‐Orp1 oxidation and stomatal aperture of individual guard cells in Col‐0 WT after control or flg22 treatment for 30 and 60 min shows that while roGFP‐Orp1 is relatively reduced in control conditions (Figure 2b), there was no correlation between stomatal aperture and roGFP2‐Orp1 oxidation (*R* = 0.07, *P* = 0.48). We found a negative correlation, albeit weak, between stomatal aperture and roGFP2‐Orp1 oxidation across all flg22 treatments (flg22 30 min, *R* = −0.24, *P* = 0.02 and flg22 60 min, *R* = −0.24, *P* = 0.01) (Figure 2b). Guard cells with roGFP2‐Orp1 in a reduced state (either control or flg22‐treated) had a wide range of apertures, whereas flg22‐treated guard cells with more oxidised roGFP2‐Orp1 all exhibited small apertures.

### RBOHF is required for PAMP‐triggered ROS production in guard cells

As a complementary approach, we used the fluorescein probe H_2_DCFDA, which is not specific to H_2_O_2_ (Murphy et al., 2022), to analyse PAMP‐induced intracellular ROS production in guard cells of NADPH oxidase mutants. H_2_DCFDA assays indicate that although the basal level of ROS was decreased in both *rbohD* and *rbohF* mutants, *rbohF* has a significantly smaller increase in ROS compared to Col‐0 by 30 min after flg22 treatment (Figure 2c). These results suggest that only RBOHF contributes to flg22‐mediated ROS production in guard cells but RBOHD does not. Importantly, based on H_2_DCFDA assays, PRX33 and PRX34 are also implicated in PAMP‐mediated ROS accumulation in guard cells (Arnaud et al., 2017). Therefore, across these mutants, ROS measurement by H_2_DCFDA shows a correlation between defects in ROS production and defects in flg22‐induced stomatal closure while, particularly in the case of *rbohF*, cytosolic roGFP2‐Orp1 is more oxidised.

### RBOHF is involved in stomatal defence responses

**Figure 3:**
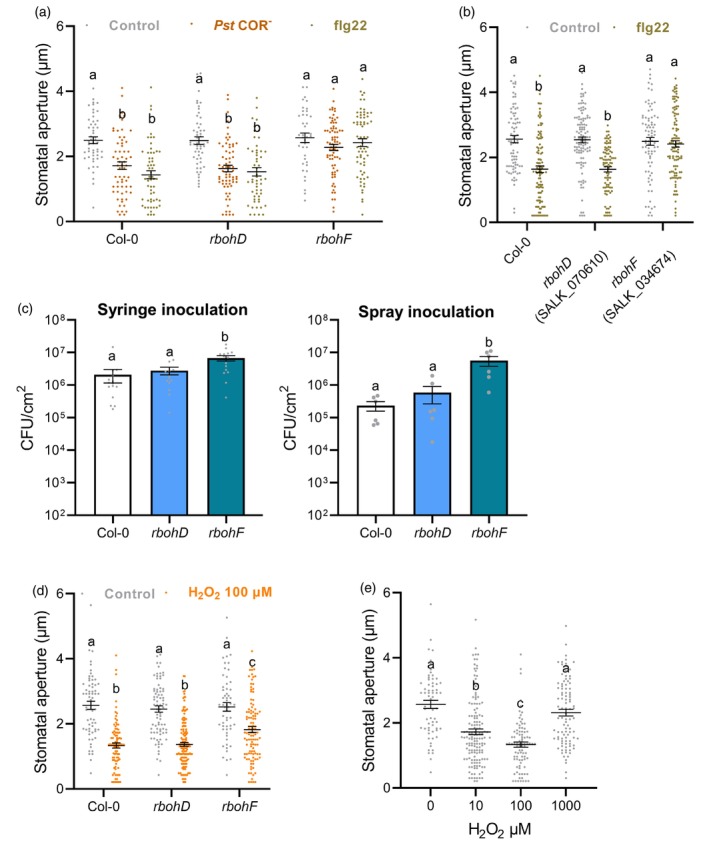
Analysis of the function of RBOHD and RBOHF during stomatal defence responses. (a) Stomatal apertures in WT Col‐0, *rbohD* and *rbohF* leaf discs exposed to mock control (10 mm MgCl_2_), 10^8^ cfu/ml COR‐deficient *Pst* DC3000 (*Pst* COR^−^) bacteria and 5 μm flg22 for 2 h. Data are means ± SE (*n* ≥ 100) from a representative experiment. (b) Stomatal apertures in Col‐0 WT and other allelic mutants of *rbohD* (SALK_070610) and *rbohF* (SALK_034674). Epidermal peels were exposed to control solution or 5 μm flg22 for 2 h. Data are means ± SE (*n* ≥ 80) from a representative experiment. (c) Bacterial growth in WT Col‐0, *rbohD* and *rbohF* mutants assessed at 3 days after syringe‐infiltration with *Pst* COR^−^ at 10^6^ cfu/ml (left panel) or after spray‐inoculation with *Pst* COR^−^ at 10^8^ cfu/ml (right panel). Values are the means ± SE (*n* = 6). (d) The *rbohF* mutant is partially defective in H_2_O_2_‐mediated stomatal closure. Stomatal apertures in WT Col‐0, *rbohD* and *rbohF* leaf discs exposed to Control or 100 μm H_2_O_2_ for 2 h. Data are means ± SE (*n* ≥ 60) from a representative experiment. (e) Dose response of Col‐0 WT stomata to H_2_O_2_ indicate that high H_2_O_2_ concentration does not close stomata. Stomatal apertures in WT Col‐0 leaf discs exposed to different concentrations of H_2_O_2_ (0, 10, 100 and 1000 μm) for 2 h. Data are means ± SE (*n* ≥ 60) from a representative experiment. Different letters indicate significant differences at *P* < 0.001 (A, B, D and E) and *P* < 0.05 (C) based on a Tukey's HSD test.

Stomatal closure is one of the first lines of defence against foliar bacteria (Melotto et al., 2006) and PAMP‐mediated stomatal closure has been correlated with increased ROS production in guard cells (Desclos‐Theveniau et al., 2012; Desikan et al., 2008). Both NADPH oxidase (RBOHD) and apoplastic peroxidases (PRX33 and PRX34) participate in stomatal immunity (Arnaud et al., 2017; Macho et al., 2012; Mersmann et al., 2010). Indeed, pre‐treatment with the non‐specific inhibitor of NADPH oxidases (DPI) or with salicylhydroxamic acid (SHAM) or sodium azide (two non‐specific inhibitors of apoplastic peroxidases) (Khokon et al., 2010) compromised flg22‐mediated stomatal closure (Figure S2a). Stomatal aperture was analysed in *rbohD* and *rbohF* leaf discs after treatment with flg22 or inoculation with the coronatine‐deficient *Pseudomonas syringae* pv. tomato DC3000 (*Pst* COR^−^) bacteria for 2 h (Figure 3a). A comparison with epidermal peels was also made (Figures S2b,c). Because the toxin coronatine counteracts PAMP‐mediated stomatal closure, the *Pst* COR^−^ strain has been widely used to characterise mutants defective in stomatal immunity (Desclos‐Theveniau et al., 2012; Kadota et al., 2014; Macho et al., 2012; Melotto et al., 2006). Contrary to previous observations showing that RBOHD is required for PAMP‐induced stomatal closure (Kadota et al., 2014; Li et al., 2014; Macho et al., 2012; Mersmann et al., 2010), we found that in our experimental conditions, *rbohD* consistently exhibited WT stomatal closure upon PAMP or bacterial treatments on both leaf disc and epidermal peel assays (Figure 3a, Figure S2b,c). On the contrary, the *rbohF* mutant was clearly defective in PAMP‐ and bacteria‐mediated stomatal closure (Figure 3a, Figure S2b,c). These results were confirmed for flg22 using different mutant alleles of these NADPH oxidases (Figure 3b). As a control, we confirmed that *rbohF*, but not *rbohD*, is partially impaired in abscisic acid‐mediated stomatal closure (Figure S3a; Kwak et al., 2003, Macho et al., 2012). Moreover, as previously observed (Khokon et al., 2010), these NADPH‐oxidases are not involved in salicylic acid‐mediated stomatal closure (Figure S3b). Although *RBOHD* is reported to be more expressed in guard cells than *RBOHF* (Kwak et al., 2003; Morales et al., 2016), RT‐qPCR measurements show that, contrary to *RBOHD*, *RBOHF* expression was induced in guard cells 2 h after flg22 treatment (Figure S2d). Compared with the PAMP‐mediated induction of *RBOHD* expression in young seedlings (Morales et al., 2016), our results indicate tissue‐specific induction of these two NADPH oxidases after PAMP activation and highlight the importance of RBOHF in stomatal immunity.

Because a defect in stomatal defence often results in enhanced susceptibility to bacteria (Melotto et al., 2006), leaves were spray‐inoculated with *Pst* COR^−^ bacteria and bacterial growth was evaluated 3 days later (Figure 3c). The *rbohF* mutant was more susceptible than the Col‐0 control to surface‐inoculated bacteria, with a 24‐fold increase in the bacterial population. As observed before (Kadota et al., 2014, Macho et al., 2012) the *rbohD* mutant was as susceptible as Col‐0 to surface‐inoculated *Pst* COR^−^ corroborating the absence of defects in bacteria‐mediated stomatal closure. To verify any deficiency in post‐invasive defences, plants were also inoculated with *Pst* COR^−^ by syringe infiltration. Similar to previous results with the WT *Pst* DC3000 bacteria (Chaouch et al., 2012), the *rbohD* mutant exhibited WT susceptibility to bacteria while *rbohF* was slightly more susceptible to syringe‐inoculated *Pst* COR^−^ with a threefold increase in bacterial titre as compared to Col‐0 (Figure 3c). These results indicate that RBOHF is important for resistance to *Pst* DC3000 bacteria and contributes more to pre‐invasive than post‐invasive defence responses.

### The *rbohF* mutant is partially defective in H_2_O_2_ ‐mediated stomatal closure

We also tested the stomatal response of *rbohD* and *rbohF* mutants to H_2_O_2_ which closes stomata through the activation of Ca^2+^ channels and inhibition of the inward K^+^ channels (Pei et al., 2000; Zhang et al., 2001). The results indicate that *rbohF* mutants were partially impaired in H_2_O_2_‐mediated stomatal closure while WT stomatal closure was observed for the *rbohD* mutants (Figure 3d, Figure S3c). Therefore, RBOHF may also act downstream of H_2_O_2_ during PAMP‐mediated stomatal closure. While H_2_O_2_ at 100 μm is widely used for inducing stomatal closure, this effect of H_2_O_2_ at this concentration is dependent upon the experimental setup (Li et al., 2021; Pei et al., 2000). Moreover, the *rbohF* mutant showed increased oxidation of the H_2_O_2_ sensor roGFP2‐Orp1 in guard cells after PAMP treatment (Figure 2a) and in whole leaf discs after H_2_O_2_ treatment (Arnaud et al., 2023). Therefore, we also tested the effect of different H_2_O_2_ concentrations on stomatal aperture in our experimental conditions using Col‐0 WT leaf discs in stomatal buffer. Surprisingly, while H_2_O_2_ at 10 and 100 μm effectively induced significant stomatal closure, we found that stomatal apertures at a higher H_2_O_2_ concentration (1 mm) were similar to the control treatment (Figure 3e). These results suggest that excess H_2_O_2_ accumulation in *rbohF* guard cells above a certain threshold is unable to induce stomatal closure.

### The *rbohF* mutant is affected in flg22‐mediated apoplastic alkalinisation

**Figure 4:**
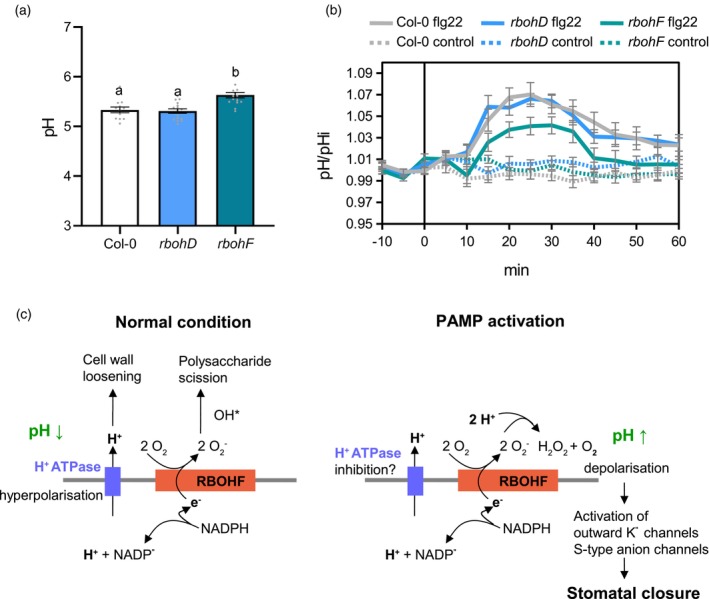
RBOHF influences apoplastic pH. (a) Apoplastic pH in Col‐0 WT, *rbohD* and *rbohF* mutant leaves. Apoplastic pH in untreated condition was determined by multiwell fluorimetry (sequential excitation at 440 ± 8 and 495 ± 8 nm; emission, 525 ± 20 nm) on Oregon green dextran‐infiltrated leaf discs from 5 week‐old plants (See Methods for details). Data are means ± SE (*n* ≥ 12) from a representative experiment. Different letters indicate significant differences at *P* < 0.001 based on a Tukey's HSD test. (b) Kinetics of flg22‐induced leaf apoplastic alkalinisation in Col‐0 WT, *rbohD* and *rbohF* mutants. Oregon green dextran‐infiltrated leaf discs were exposed at *t* = 0 min to control solution or 1 μm flg22, the apoplastic pH was measured over time by multiwell fluorimetry and expressed relative to the mean initial pH (pHi) before treatment (pH/pHi). Data are means ± SE (*n* ≥ 18) from 3 independent experiments. Significant differences at *P* < 0.05 between flg22‐treated Col‐0 and *rbohF* were found between 10 and 60 min based on two‐way ANOVA and uncorrected Fisher's LSD analyses for each time point. (c) Hypothetical model of the regulation of apoplastic pH by RBOHF during PTI activation. In normal unstressed condition, the efflux of electron (e^−^) produced by the NADPH oxidase activity is compensated by an efflux of proton (H^+^) probably generated by plasma membrane H + ATPases. Both the acidification of the apoplast and the production of hydroxyl radicals (OH^•^) from superoxide (O_2_^−^) through the Haber–Weiss and Fenton reactions contribute to cell expansion. The plant immune response may induce the inhibition of H^+^ATPases and the activation of RBOHF together with superoxide dismutases (SOD) or germin‐like proteins which dismutates O_2_^−^ to H_2_O_2_, a reaction that consumes H^+^. The resulting alkalinisation of the apoplast induces a depolarisation of the plasma membrane ultimately leading to stomatal closure via the activation of ion channels.

Apoplastic alkalinisation is a well‐characterised early event induced upon PAMP perception (Felix et al., 1999; Felle et al., 2004) and constitutively active plasma membrane H^+^ATPase OST2/AHA1 impedes PAMP‐mediated stomatal closure and apoplastic ROS burst (Keinath et al., 2010; Liu et al., 2009). Otte and collaborators showed that elicitor‐induced alkalinisation of the apoplast was inhibited by DPI in chickpea (Otte et al., 2001) and NADPH oxidases could contribute to extracellular pH changes through their electrogenic activities and/or the consumption of protons during superoxide dismutation (Segal, 2016). To test this hypothesis, we used the ratiometric Oregon green fluorescent dye which has been successfully used in Arabidopsis (Geilfus & Muehling, 2011; McLachlan et al., 2016) and adapted the method for multi‐well fluorimetry on leaf discs. Interestingly, assays with Oregon green indicate that the apoplastic pH was higher in the *rbohF* mutant in unstressed conditions while it remained unchanged in the *rbohD* background (Figure 4a). This result nicely correlates with the impaired growth of *rbohF* rosette leaves observed previously (Chaouch et al., 2012; Torres et al., 2002) and in our laboratory conditions suggesting that RBOHF is important for the acidic growth of plant cells. Although *rbohD* and *rbohF* showed WT increase in absolute pH after flg22 treatment (Figure S3d), the flg22‐triggered increase in pH relative to the initial pH in unstressed conditions was significantly lower in the *rbohF* mutant compared to Col‐0 WT (Figure 4b). These results suggest that the *rbohF* mutant is partly defective in PAMP‐induced apoplastic alkalinisation. Therefore, as observed for the constitutive active *ost2‐1D* and *ost2‐2D* mutants (Liu et al., 2009; Merlot et al., 2007), defects in apoplastic alkalinisation may also contribute to the impairment of *rbohF* in H_2_O_2_‐, ABA‐ and PAMP‐mediated stomatal closure.

## DISCUSSION

### The role of RBOHF in stomatal defences

PAMP‐mediated stomatal closure is considered to involve ROS production (Arnaud & Hwang, 2015). Previous reports suggest that the RBOHD isoform is required for closure, presumably mediated by ROS production initially in the apoplast (Kadota et al., 2014; Li et al., 2014; Macho et al., 2012; Mersmann et al., 2010). However, the defect in PAMP‐mediated stomatal closure observed in the *rbohD* mutant was not correlated with increased susceptibility to *Pst* bacteria (Kadota et al., 2014; Macho et al., 2012). A more recent study showed that RBOHD and RBOHF are not involved in flg22‐triggered stomatal closure (Guzel Deger et al., 2015). Contrary to previous work, and using multiple mutant alleles, we found that stomatal closure in response to flg22 and to *Pst* COR^−^ bacteria depends on RBOHF but not on RBOHD. Correspondingly, there was increased bacterial growth in the *rbohF* mutant but not *rbohD*. It should be noted that the *rbohD* and *rbohF* mutants showed the expected responses for the apoplastic burst in response to flg22. Our results and previous data suggest that the relative contribution of RBOHD and RBOHF in stomatal immunity likely depends on experimental conditions. While unquantified differences in growth conditions may be involved, it should also be noted that immune responses are strongly dependent on the circadian clock which could, for example, mean that different NADPH oxidase isoforms influence responses at different times of day (Zhang et al., 2013).

### Is cytosolic H_2_O_2_ important for stomatal closure?

The relationship between ROS production and stomatal closure was complex. The widely used probe H_2_DCFDA, which it is not specific for any particular ROS and not oxidised directly by H_2_O_2_ (Murphy et al., 2022) showed decreased ROS production in *rbohF* guard cells in response to flg22, which is commensurate with defects in stomatal closure. roGFP2‐Orp1 and GRX1‐roGFP2 have been used to demonstrate oxidation in guard cell cytosol during NaHS‐induced stomatal closure (Scuffi et al., 2018). However, during the flg22 response, roGFP2‐Orp1 oxidation state did not show a strong relationship with the aperture and the probe was more oxidised in *rbohF* guard cells despite a lower H_2_DCFDA oxidation. As suggested previously for whole leaves (Arnaud et al., 2023; Chaouch et al., 2012), a lower antioxidant capacity in *rbohF* guard cells may explain the higher roGFP2‐Orp1 oxidation upon PAMP activation. This could reduce the thiol‐reducing capacity affecting roGFP2‐Orp1 regeneration without influencing H_2_DCFDA oxidation. Added to this, stomatal aperture in Col‐0 had a poor correlation with roGFP2‐Orp1 oxidation state in control conditions. Using epidermal strips incubated in stomatal opening buffer, the stomatal aperture seems not to be affected by high roGFP2‐Orp1 oxidation in guard cells (Scuffi et al., 2018). A possible explanation is that roGFP2‐Orp1 is specifically reporting cytosolic H_2_O_2_ which could be derived from multiple compartments. For example, mitochondrial ROS contribute to ABA‐mediated stomatal closure (Postiglione & Muday, 2023). The use of catalase mutants has shown that, despite higher intracellular H_2_O_2_, stomatal aperture is unaffected although still responsive to exogenous H_2_O_2_ (Jannat et al., 2011). This may explain why stomata remain open despite photosynthetic ROS production in the light. Our results, therefore, support the uncoupling of cytosolic H_2_O_2_ from stomatal aperture and that the extracellular production of superoxide or H_2_O_2_ by RBOHF is critical for flg22‐induced closure. The LRR receptor kinase HPCA1 is a proposed extracellular H_2_O_2_ sensor which, when oxidised on extracellular cysteines, activates calcium channels leading to increased intracellular Ca^2+^ (Wu et al., 2020). This sensor provides an explanation for the requirement of apoplastic H_2_O_2_ for stomatal closure. Intriguingly, our results suggest that the ROS probe H_2_DCFDA could indicate that ROS other than H_2_O_2_ are involved in triggering closure while roGFP2‐Orp1 indicates cytosolic conditions that are at least partly independent of stomatal aperture. The results with guard cells therefore partly mirror the recent findings with whole leaves (Arnaud et al., 2023) where the RBOHD‐dependent apoplastic oxidative burst measured by luminol is independent of subsequent prolonged oxidation of cytosolic roGFP2‐Orp1.

### Putative role of RBOHF in PAMP‐mediated apoplastic alkalinisation

The *rbohF* mutant was affected in stomatal closure induced by exogenous H_2_O_2_ suggesting that, besides ROS production, RBOHF also functions in other mechanisms important for stomatal immunity. Interestingly, despite a higher apoplastic pH under normal conditions, the *rbohF* mutant was affected in flg22‐induced apoplastic alkalinisation. In the *rbohC* mutant, the amplitude of apoplastic pH fluctuations during root hair elongation is affected (Monshausen et al., 2007). It was shown recently that damage‐induced membrane depolarisation in roots is impaired in *rbohD* and *rbohF* mutants (Marhavý et al., 2019) and RBOHD might be important in propagating the systemic electric potential induced by wounding in leaves (Suzuki et al., 2013). These results suggest that besides superoxide production, plant NADPH oxidases like their animal counterparts (Segal, 2016) are electrogenic through electron transport across the plasma membrane which is coupled to proton efflux probably through H^+^‐ATPases (Figure 4c). These extracellular H^+^ ions could participate in cell wall loosening *via* the expansion activities in normal conditions (Cosgrove, 2005). During PAMP‐triggered immunity, superoxide dismutation to H_2_O_2_, probably catalysed by unknown PAMP‐activated superoxide dismutases or germin‐like proteins (Smirnoff & Arnaud, 2019), consumes protons and could increase apoplastic pH (Figure 4c) which in turn activates apoplastic peroxidases (Bolwell et al., 2002). Alternatively, *rbohF* could have an indirect effect on the activity of unknown proton transporters or H^+^‐ATPases at the plasma membrane (Segal, 2016). It has been suggested that flg22 inhibits AHA1 and AHA2 H^+^‐ATPase *via* dephosphorylation (Elmore & Coaker, 2011; Nuhse et al., 2007). More importantly, apoplastic alkalinisation driven by RBOHF or AHA1/2 inhibition likely contributes to plasma membrane depolarisation which is well known to activate outward K^+^ channels and S‐type anion channels leading to stomatal closure (Kollist et al., 2014). The function of RBOHF, RBOHD and other NADPH oxidases in connecting extra‐ and intracellular ROS production, apoplastic and cytosolic pH changes and plasma membrane polarisation during plant response to environmental stresses needs to be further explored.

## EXPERIMENTAL PROCEDURES

### Statistical analysis

The experiments reported here were repeated at least three times with similar results unless otherwise mentioned. Experiments were analysed by Student's *t*‐tests or ANOVAs followed by Tukey's honestly significant difference (HSD) post hoc test using R software (R Core Team, https://www.R‐project.org). Raw data for stomatal aperture measurements are in Supplemental Table S1. The time course experiment was analysed by 2‐way ANOVA. Significant differences between each treatment at each time were determined by uncorrected Fisher's LSD test using GraphPad Prism v8 (GraphPad, San Diego, California, USA).

### Plant materials and growth conditions

The roGFP2‐Orp1 line (Nietzel et al., 2019) and all mutant lines are in Arabidopsis Col‐0 background. *rbohD* (CS9555), *rbohF* (CS9557), *prx33‐3* (GK‐014E05) and *prx34‐2* (GK‐728F08), were previously described (Arnaud et al., 2017; Torres et al., 2002). The *rbohF* (SALK_034674) and *rbohD* (SALK_070610) lines were obtained from the European Arabidopsis Stock Centre. All T‐DNA insertion mutants were confirmed by PCR genotyping prior to use (Supplemental Table S2). The genotyping of *rboh* mutants is shown in Figures S4 and S5. The obtention of roGFP2‐Orp1 *rbohD*, roGFP2‐Orp1 *rbohF*, roGFP2‐Orp1 *prx33‐3*, roGFP2‐Orp1 *prx34‐2* lines were previously described (Arnaud et al., 2023). Four‐ to 5‐week‐old plants grown on soil in a growth chamber under short‐day conditions (10 h light at 22°C/14 h dark at 19°C), at 60% humidity and illuminated with fluorescent tubes at 100 μmol m^−2^ sec^−1^ light intensity were used for all the experiments. For the experiments described below, treatments with chemicals or bacteria were performed 5–6 h after the light switch was on.

### Chemicals

Purified chemicals, except the flg22 peptide (Peptron, Korea), were purchased from Sigma. Control solutions were stomatal buffer containing 1% ethanol for 2 mm salicylhydroxamic acid (SHAM), 0.1% DMSO for 20 μm diphenyleneiodonium chloride (DPI) and water for 1 μm sodium azide, 10 μm to 1 mm hydrogen peroxide (H_2_O_2_), 5 μm flg22.

### Bacterial infection assay

The bacterial strain *Pst* DC3000 COR^−^ (DB4G3) (Brooks et al., 2004) was cultivated overnight at 28°C in King's B medium supplemented with Kanamycin and Rifampicin (each at 100 μg/ml). Bacteria were collected by centrifugation at 3000 ***g*** for 5 min at room temperature and washed twice in 10 mm MgCl_2_. Plants were surface‐inoculated by spraying with a bacterial solution of 10^8^ cfu/ml in 10 mm MgCl_2_ containing 0.02% Silwet L‐77, and plants were covered to maintain high humidity until disease symptoms developed. Alternatively, rosette leaves were syringe‐infiltrated with a bacterial solution of 10^6^ cfu/ml in 10 mm MgCl_2_. After 3 days, bacterial growth in the apoplast of three leaves per plant and six plants per genotype was determined as previously described (Katagiri et al., 2002).

### Luminol assay

Leaf discs of 6 mm diameter were cut into four equal pieces, immersed in distilled water and incubated for 3 h minimum at room temperature for recovery after wounding. Before starting the assay, water was exchanged by a solution containing 100 μm luminol and 10 μg/ml horseradish peroxidase (≥ 250 units/mg solid). After adding a control solution or 1 μm flg22, the luminescence was measured immediately using a CLARIOstar plate reader (BMG Labtech, Aylesbury, UK) with a reading time of 2 sec.

### Confocal imaging and image analysis

Leaf discs from rosette leaves were immersed in 10 mm MES‐KOH pH 6.15, 30 mM KCl, incubated for 2 h at 21°C under laboratory lighting (PPFD ~10 μmol m^−2^ sec^−1^) for recovery after wounding and subsequently treated with control solution or 1 μm flg22 for 30 and 60 min. Leaf discs were mounted under a Zeiss confocal microscope LSM 880. Images were collected with a 20X lens (Plan‐Apochromat, 0.8 numerical aperture) and roGFP2‐Orp1 was excited sequentially at 405 and 488 nm and emission was detected at 508–526 nm, with the pinhole set to 1 airy unit. Single‐plane images were processed with ImageJ software. Background fluorescence was insignificant and not subtracted. Fluorescence intensity values of individual guard cells selected as region of interest were quantified on 32‐bit converted images and the ratio 405/488 nm was calculated.

### Measurements of stomatal aperture

Stomatal experiments were conducted as previously described (Desclos‐Theveniau et al., 2012). Epidermal peels collected from the abaxial side of young fully expanded leaves or leaf discs were floated in stomatal buffer (10 mm MES‐KOH pH 6.15, 30 mm KCl) for 2.5 h under light (100 μmol m^−2^ sec^−1^) to ensure that most stomata were opened before treatments. Solutions of chemicals or bacterial suspension at 10^8^ cfu.ml^−1^ in 10 mm MgCl_2_ were directly added to the stomatal buffer. After treatments, epidermal peels or leaf discs were further incubated under light for 2 h and observed under a light microscope (Carl Zeiss, Axioplan 2). Stomatal apertures of stomata in random areas were measured using ImageJ 1.42 software. Measurements of stomatal aperture were performed 7–8 h after starting illumination.

### Monitoring ROS in guard cells

2′,7′ dichl orofluorescein diacetate (H_2_DCFDA, Sigma) was used to measure ROS in guard cells (Pei et al., 2000). After 2.5 h incubation in stomatal buffer, epidermal peels were incubated with 50 μm H_2_DCFDA in 0.1% DMSO for 15 min. Excess H_2_DCFDA was then removed by washing 3 times for 20 min with stomatal buffer. Then, 5 μm flg22 or control solution was added. After 30 min incubation, H_2_DCFDA fluorescence was observed with a fluorescence microscope (Carl Zeiss, Axioplan 2) and fluorescence intensity of guard cells was analysed using ImageJ software.

### Gene expression analysis in guard cell protoplasts

For each condition, about 50 young fully expanded leaves were immerged for 2 h in stomatal buffer containing 0.02% (v/v) Silwet‐L77 with or without 1 μm flg22. Guard cell protoplasts were isolated as previously described (Obulareddy et al., 2013) in the presence of transcriptional inhibitors 0.01% (w/v) cordycepin and 0.0033% (w/v) actinomycin D. For each condition, about 10^6^ guard cell protoplasts were obtained and their purity was above 98%. RNA was extracted using the RNeasy Plant Mini Kit with in‐column Dnase 1 digestion (Qiagen, Hilden, Germany). Two hundred nanograms of total RNA were reverse transcribed using 500 ng of oligo(dT)15 and the ImProm‐II Reverse Transcription System following the manufacturer's instructions (Promega, Madison, WI, USA). Quantitative real‐time PCR reaction was performed on a Light Cycler 2 (Roche, Basel, Switzerland) using 10 μl SYBR Premix Ex Taq (Takara, Kusatsu, Japan), 2 μl of twofold diluted cDNA and 0.5 μm of primers in a total volume of 20 μl per reaction. The cycling conditions were composed of an initial 20 sec denaturation step at 95°C, followed by 45 cycles of 95°C for 7 sec, 60°C for 10 sec and 72°C for 13 sec. A melting curve was run from 65°C to 95°C to ensure the specificity of the products. Data were analysed with the delta Ct method. Ubiquitin 1 (*UBQ1*) was used as a reference gene for normalisation of gene expression levels. Control treatment was considered as expression level = 1. As controls, the expression of the reference gene *ACT2* did not change between control and flg22 conditions while the expression of the PTI marker gene *PRX4* was induced by flg22 (Figure S2d). qRT‐PCR primer sequences are listed in Supplemental Table S2.

### Ratiometric pH quantification using Oregon green

Fully expanded leaves were syringe‐infiltrated with 25 μm of Oregon Green 488 dextran (ThermoFisher, Waltham, MA, USA) (Geilfus & Muehling, 2011; McLachlan et al., 2016). For the autofluorescence background, milliQ water was infiltrated into the apoplast. Plants were kept in the growth chamber for 2 h until excess water has evaporated. Oregon Green‐treated leaf discs were placed in a 96‐well plate, immersed in milliQ water and further incubated for 2 h at 21°C under laboratory lighting (PPFD ~10 μmol m^−2^ sec^−1^) for recovery after wounding. Oregon Green was excited sequentially at 440 nm ± 8 nm and 495 nm ± 8 nm in a CLARIOstar plate reader (BMG Labtech) and emission was recorded at 525 nm ± 20 nm with a gain set at 1250 and 1000 for the 440 and 495 nm excitations, respectively. Each leaf disc was scanned from the top with the fluorescence recorded and averaged from 76 flashes per well‐organised as a spiral of 5 mm diameter. The initial 495/440 ratio of the resting state of leaf discs was estimated by reading the wells for 15 min before treatment. The emission of water‐infiltrated leaf discs was averaged and subtracted for all the data points to correct for background fluorescence. To convert the fluorescence 495/440 ratio into pH values, a calibration curve with pH‐buffered Oregon Green solution infiltrated into the apoplast of leaves was performed according to (McLachlan et al., 2016). The Boltzmann fit was chosen for fitting sigmoidal curves to calibration data and pH was determined according to the equation:

$$\;x=V50-\text{Slope In}\;\left(\frac{\;\mathrm{Top}-y}{y-\text{Bottom}}\right)$$

where *x* = pH, *y* = 495/440 ratio, Bottom = 0.4833, Top = 2.045, V50 = 4.513 and Slope = 0.6267. Changes in pH over time were normalised to the initial pHi as pH/pHi.

## ACCESSION NUMBERS

The *A*. *thaliana* genes included in this study are as follows: *PRX33* (At3g49110); *PRX34* (At3g49120); *RDOHD* (At5g47910); and *RBOHF* (At1g64060).

## AUTHOR CONTRIBUTIONS

DA designed and performed the research, analysed the data and wrote the paper. MJD designed the research, analysed the data and wrote the paper. NS designed the research, analysed the data and wrote the paper.

## CONFLICT OF INTEREST

The authors declare no conflict of interest.

### OPEN RESEARCH BADGES

This article has earned an Open Data badge for making publicly available the digitally‐shareable data necessary to reproduce the reported results. The data is available.

## Supporting information


**Figure S1.** Flg22‐triggered roGFP2‐Orp1 oxidation in pavement cells and guard cells.
**Figure S2.** The NADPH oxidase RBOHF activates stomatal immunity.
**Figure S3.** The *rbohF* mutant is partly defective in ABA‐ and H_2_O_2_‐mediated stomatal closure.
**Figure S4.** Genotyping by PCR of *rbohD* and *rbohF* dSpm transposon mutants.
**Figure S5.** Genotyping by PCR of *rbohD* and *rbohF* Salk T‐DNA mutants.

**Table S1.** Raw data of stomatal aperture measurements (in μm) involving the *rbohD* and *rbohF* mutants.

**Table S2.** PCR primers used for genotyping and RT‐qPCR.

## References

[tpj16380-bib-0001] Arnaud, D. , Deeks, M.J. & Smirnoff, N. (2023) Organelle‐targeted biosensors reveal distinct oxidative events during pattern‐triggered immune responses. Plant Physiology, 191, 2551–2569. 36582183 10.1093/plphys/kiac603 PMC10069903

[tpj16380-bib-0002] Arnaud, D. & Hwang, I. (2015) A sophisticated network of signaling pathways regulates stomatal defenses to bacterial pathogens. Molecular Plant, 8, 566–581. 25661059 10.1016/j.molp.2014.10.012

[tpj16380-bib-0003] Arnaud, D. , Lee, S. , Takebayashi, Y. , Choi, D. , Choi, J. , Sakakibara, H. et al. (2017) Cytokinin‐mediated regulation of reactive oxygen species homeostasis modulates stomatal immunity in Arabidopsis. Plant Cell, 29, 543–559. 28254779 10.1105/tpc.16.00583 PMC5385949

[tpj16380-bib-0004] Bolwell, G.P. , Bindschedler, L.V. , Blee, K.A. , Butt, V.S. , Davies, D.R. , Gardner, S.L. et al. (2002) The apoplastic oxidative burst in response to biotic stress in plants: a three‐component system. Journal of Experimental Botany, 53, 1367–1376. 11997382

[tpj16380-bib-0005] Brooks, D.M. , Hernandez‐Guzman, G. , Kloek, A.P. , Alarcon‐Chaidez, F. , Sreedharan, A. , Rangaswamy, V. et al. (2004) Identification and characterization of a well‐defined series of coronatine biosynthetic mutants of *Pseudomonas syringae* pv. *Tomato* DC3000. Molecular Plant‐Microbe Interactions, 17, 162–174. 14964530 10.1094/MPMI.2004.17.2.162

[tpj16380-bib-0006] Chaouch, S. , Queval, G. & Noctor, G. (2012) AtRbohF is a crucial modulator of defence‐associated metabolism and a key actor in the interplay between intracellular oxidative stress and pathogenesis responses in Arabidopsis. Plant Journal, 69, 613–627. 10.1111/j.1365-313X.2011.04816.x 21985584

[tpj16380-bib-0007] Cosgrove, D.J. (2005) Growth of the plant cell wall. Nature Reviews Molecular Cell Biology, 6, 850–861. 16261190 10.1038/nrm1746

[tpj16380-bib-0008] Daudi, A. , Cheng, Z. , O'Brien, J.A. , Mammarella, N. , Khan, S. , Ausubel, F.M. et al. (2012) The apoplastic oxidative burst peroxidase in *Arabidopsis* is a major component of pattern‐triggered immunity. Plant Cell, 24, 275–287. 22247251 10.1105/tpc.111.093039 PMC3289579

[tpj16380-bib-0009] Desclos‐Theveniau, M. , Arnaud, D. , Huang, T.‐Y. , Lin, G.J.‐C. , Chen, W.‐Y. , Lin, Y.‐C. et al. (2012) The Arabidopsis lectin receptor kinase LecRK‐V.5 represses stomatal immunity induced by *Pseudomonas syringae* pv. *Tomato* DC3000. PLoS Pathogens, 8, e1002513. 22346749 10.1371/journal.ppat.1002513 PMC3276567

[tpj16380-bib-0010] Desikan, R. , Horak, J. , Chaban, C. , Mira‐Rodado, V. , Witthoft, J. , Elgass, K. et al. (2008) The histidine kinase AHK5 integrates endogenous and environmental signals in Arabidopsis guard cells. PLoS One, 3, e2491. 18560512 10.1371/journal.pone.0002491 PMC2424244

[tpj16380-bib-0011] Elmore, J.M. & Coaker, G. (2011) The role of the plasma membrane H^+^‐ATPase in plant–microbe interactions. Molecular Plant, 4, 416–427. 21300757 10.1093/mp/ssq083 PMC3107590

[tpj16380-bib-0012] Felix, G. , Duran, J.D. , Volko, S. & Boller, T. (1999) Plants have a sensitive perception system for the most conserved domain of bacterial flagellin. Plant Journal, 18, 265–276. 10.1046/j.1365-313x.1999.00265.x 10377992

[tpj16380-bib-0013] Felle, H.H. , Herrmann, A. , Hanstein, S. , Hückelhoven, R. & Kogel, K.‐H. (2004) Apoplastic pH signaling in barley leaves attacked by the powdery mildew fungus *Blumeria graminis* f. sp. *hordei* . Molecular Plant‐Microbe Interactions, 17, 118–123. 14714875 10.1094/MPMI.2004.17.1.118

[tpj16380-bib-0014] Geilfus, C.‐M. & Muehling, K. (2011) Real‐time imaging of leaf apoplastic pH dynamics in response to NaCl stress. Frontiers in Plant Science, 2, 13. 22639578 10.3389/fpls.2011.00013 PMC3355670

[tpj16380-bib-0015] Guzel Deger, A. , Scherzer, S. , Nuhkat, M. , Kedzierska, J. , Kollist, H. , Brosché, M. et al. (2015) Guard cell SLAC1‐type anion channels mediate flagellin‐induced stomatal closure. New Phytologist, 208, 162–173. 25932909 10.1111/nph.13435 PMC4949714

[tpj16380-bib-0016] Jannat, R. , Uraji, M. , Morofuji, M. , Islam, M.M. , Bloom, R.E. , Nakamura, Y. et al. (2011) Roles of intracellular hydrogen peroxide accumulation in abscisic acid signaling in Arabidopsis guard cells. Journal of Plant Physiology, 168, 1919–1926. 21665322 10.1016/j.jplph.2011.05.006 PMC4073789

[tpj16380-bib-0017] Kadota, Y. , Sklenar, J. , Derbyshire, P. , Stransfeld, L. , Asai, S. , Ntoukakis, V. et al. (2014) Direct regulation of the NADPH oxidase RBOHD by the PRR‐associated kinase BIK1 during plant immunity. Molecular Cell, 54, 43–55. 24630626 10.1016/j.molcel.2014.02.021

[tpj16380-bib-0018] Katagiri, F. , Thilmony, R. & He, S.Y. (2002) The *Arabidopsis thaliana*‐*Pseudomonas syringae* interaction. In: The Arabidopsis book. Washington DC: BioOne, pp. e0039. 10.1199/tab.0039 PMC3243347 22303207

[tpj16380-bib-0019] Keinath, N.F. , Kierszniowska, S. , Lorek, J. , Bourdais, G. , Kessler, S.A. , Shimosato‐Asano, H. et al. (2010) PAMP (pathogen‐associated molecular pattern)‐induced changes in plasma membrane compartmentalization reveal novel components of plant immunity. Journal of Biological Chemistry, 285, 39140–39149. 20843791 10.1074/jbc.M110.160531 PMC2998143

[tpj16380-bib-0020] Khokon, M.A.R. , Hossain, M.A. , Munemasa, S. , Uraji, M. , Nakamura, Y. , Mori, I.C. et al. (2010) Yeast elicitor‐induced stomatal closure and peroxidase‐mediated ROS production in Arabidopsis. Plant and Cell Physiology, 51, 1915–1921. 20876608 10.1093/pcp/pcq145

[tpj16380-bib-0021] Kollist, H. , Nuhkat, M. & Roelfsema, M.R.G. (2014) Closing gaps: linking elements that control stomatal movement. New Phytologist, 203, 44–62. 24800691 10.1111/nph.12832

[tpj16380-bib-0022] Kwak, J.M. , Mori, I.C. , Pei, Z.‐M. , Leonhardt, N. , Torres, M.A. , Dangl, J.L. et al. (2003) NADPH oxidase *AtrbohD* and *AtrbohF* genes function in ROS‐dependent ABA signaling in Arabidopsis. The EMBO Journal, 22, 2623–2633. 12773379 10.1093/emboj/cdg277 PMC156772

[tpj16380-bib-0023] Li, K. , Prada, J. , Damineli, D.S.C. , Liese, A. , Romeis, T. , Dandekar, T. et al. (2021) An optimized genetically encoded dual reporter for simultaneous ratio imaging of Ca^2+^ and H^+^ reveals new insights into ion signaling in plants. New Phytologist, 230, 2292–2310. 33455006 10.1111/nph.17202 PMC8383442

[tpj16380-bib-0024] Li, L. , Li, M. , Yu, L. , Zhou, Z. , Liang, X. , Liu, Z. et al. (2014) The FLS2‐associated kinase BIK1 directly phosphorylates the NADPH oxidase RbohD to control plant immunity. Cell Host & Microbe, 15, 329–338. 24629339 10.1016/j.chom.2014.02.009

[tpj16380-bib-0025] Liu, J. , Elmore, J.M. , Fuglsang, A.T. , Palmgren, M.G. , Staskawicz, B.J. & Coaker, G. (2009) RIN4 functions with plasma membrane H^+^‐ATPases to regulate stomatal apertures during pathogen attack. PLoS Biology, 7, e1000139. 19564897 10.1371/journal.pbio.1000139 PMC2694982

[tpj16380-bib-0026] Macho, A.P. , Boutrot, F. , Rathjen, J.P. & Zipfel, C. (2012) ASPARTATE OXIDASE plays an important role in Arabidopsis stomatal immunity. Plant Physiology, 159, 1845–1856. 22730426 10.1104/pp.112.199810 PMC3425217

[tpj16380-bib-0027] Marhavý, P. , Kurenda, A. , Siddique, S. , Dénervaud Tendon, V. , Zhou, F. , Holbein, J. et al. (2019) Single‐cell damage elicits regional, nematode‐restricting ethylene responses in roots. The EMBO Journal, 38, e100972. 31061171 10.15252/embj.2018100972 PMC6518030

[tpj16380-bib-0028] McLachlan, D.H. , Lan, J. , Geilfus, C.M. , Dodd, A.N. , Larson, T. , Baker, A. et al. (2016) The breakdown of stored triacylglycerols is required during light‐induced stomatal opening. Current Biology, 26, 707–712. 26898465 10.1016/j.cub.2016.01.019 PMC4791430

[tpj16380-bib-0029] Melotto, M. , Underwood, W. & He, S.Y. (2008) Role of stomata in plant innate immunity and foliar bacterial diseases. Annual Review of Phytopathology, 46, 101–122. 10.1146/annurev.phyto.121107.104959 PMC2613263 18422426

[tpj16380-bib-0030] Melotto, M. , Underwood, W. , Koczan, J. , Nomura, K. & He, S.Y. (2006) Plant stomata function in innate immunity against bacterial invasion. Cell, 126, 969–980. 16959575 10.1016/j.cell.2006.06.054

[tpj16380-bib-0031] Merlot, S. , Leonhardt, N. , Fenzi, F. , Valon, C. , Costa, M. , Piette, L. et al. (2007) Constitutive activation of a plasma membrane H(+)‐ATPase prevents abscisic acid‐mediated stomatal closure. The EMBO Journal, 26, 3216–3226. 17557075 10.1038/sj.emboj.7601750 PMC1914098

[tpj16380-bib-0032] Mersmann, S. , Bourdais, G. , Rietz, S. & Robatzek, S. (2010) Ethylene signaling regulates accumulation of the FLS2 receptor and is required for the oxidative burst contributing to plant immunity. Plant Physiology, 154, 391–400. 20592040 10.1104/pp.110.154567 PMC2938167

[tpj16380-bib-0033] Monshausen, G.B. , Bibikova, T.N. , Messerli, M.A. , Shi, C. & Gilroy, S. (2007) Oscillations in extracellular pH and reactive oxygen species modulate tip growth of Arabidopsis root haris. Proceedings of the National Academy of Sciences of the United States of America, 104, 20996–21001. 18079291 10.1073/pnas.0708586104 PMC2409255

[tpj16380-bib-0034] Morales, J. , Kadota, Y. , Zipfel, C. , Molina, A. & Torres, M.‐A. (2016) The Arabidopsis NADPH oxidases RbohD and RbohF display differential expression patterns and contributions during plant immunity. Journal of Experimental Botany, 67, 1663–1676. 26798024 10.1093/jxb/erv558

[tpj16380-bib-0035] Murphy, M.P. , Bayir, H. , Belousov, V. , Chang, C.J. , Davies, K.J.A. , Davies, M.J. et al. (2022) Guidelines for measuring reactive oxygen species and oxidative damage in cells and in vivo. Nature Metabolism, 4, 651–662. 10.1038/s42255-022-00591-z PMC9711940 35760871

[tpj16380-bib-0036] Nietzel, T. , Elsässer, M. , Ruberti, C. , Steinbeck, J. , Ugalde, J.M. , Fuchs, P. et al. (2019) The fluorescent protein sensor roGFP2‐Orp1 monitors in vivo H_2_O_2_ and thiol redox integration and elucidates intracellular H_2_O_2_ dynamics during elicitor‐induced oxidative burst in Arabidopsis. New Phytologist, 221, 1649–1664. 30347449 10.1111/nph.15550

[tpj16380-bib-0037] Nuhse, T.S. , Bottrill, A.R. , Jones, A.M.E. & Peck, S.C. (2007) Quantitative phosphoproteomic analysis of plasma membrane proteins reveals regulatory mechanisms of plant innate immune responses. Plant Journal, 51, 931–940. 10.1111/j.1365-313X.2007.03192.x PMC2156193 17651370

[tpj16380-bib-0038] Obulareddy, N. , Panchal, S. & Melotto, M. (2013) Guard cell purification and RNA isolation suitable for high‐throughput transcriptional analysis of cell‐type responses to biotic stresses. Molecular Plant Microbe Interactions, 26, 844–849. 23634837 10.1094/MPMI-03-13-0081-TA PMC3982617

[tpj16380-bib-0039] Otte, O. , Pachten, A. , Hein, F. & Barz, W. (2001) Early elicitor‐induced events in chickpea cells: functional links between oxidative burst, sequential occurrence of extracellular alkalinisation and acidification, K+/H+ exchange and defence‐related gene activation. Zeitschrift für Naturforschung C, 56, 65–76. 10.1515/znc-2001-1-212 11302217

[tpj16380-bib-0040] Pei, Z.M. , Murata, Y. , Benning, G. , Thomine, S. , Klusener, B. , Allen, G.J. et al. (2000) Calcium channels activated by hydrogen peroxide mediate abscisic acid signalling in guard cells. Nature, 406, 731–734. 10963598 10.1038/35021067

[tpj16380-bib-0041] Postiglione, A.E. & Muday, G.K. (2023) Abscisic acid increases hydrogen peroxide in mitochondria to facilitate stomatal closure. Plant Physiology, 192, 469–487. 36573336 10.1093/plphys/kiac601 PMC10152677

[tpj16380-bib-0042] Rodrigues, O. , Reshetnyak, G. , Grondin, A. , Saijo, Y. , Leonhardt, N. , Maurel, C. et al. (2017) Aquaporins facilitate hydrogen peroxide entry into guard cells to mediate ABA‐ and pathogen‐triggered stomatal closure. Proceedings of the National Academy of Sciences of the United States of America, 114, 9200–9205. 28784763 10.1073/pnas.1704754114 PMC5576802

[tpj16380-bib-0043] Scuffi, D. , Nietzel, T. , Di Fino, L.M. , Meyer, A.J. , Lamattina, L. , Schwarzlander, M. et al. (2018) Hydrogen sulfide increases production of NADPH oxidase‐dependent hydrogen peroxide and phospholipase D‐derived phosphatidic acid in guard cell signaling. Plant Physiology, 176, 2532–2542. 29438048 10.1104/pp.17.01636 PMC5841699

[tpj16380-bib-0044] Segal, A.W. (2016) NADPH oxidases as electrochemical generators to produce ion fluxes and turgor in fungi, plants and humans. Open Biology, 6, 160028. 27249799 10.1098/rsob.160028 PMC4892433

[tpj16380-bib-0045] Smirnoff, N. & Arnaud, D. (2019) Hydrogen peroxide metabolism and functions in plants. New Phytologist, 221, 1197–1214. 30222198 10.1111/nph.15488

[tpj16380-bib-0046] Suzuki, N. , Miller, G. , Salazar, C. , Mondal, H.A. , Shulaev, E. , Cortes, D.F. et al. (2013) Temporal‐spatial interaction between reactive oxygen species and abscisic acid regulates rapid systemic acclimation in plants. Plant Cell, 25, 3553–3569. 24038652 10.1105/tpc.113.114595 PMC3809549

[tpj16380-bib-0047] Tian, S. , Wang, X. , Li, P. , Wang, H. , Ji, H. , Xie, J. et al. (2016) Plant aquaporin AtPIP1;4 links apoplastic H_2_O_2_ induction to disease immunity pathways. Plant Physiology, 171, 1635–1650. 26945050 10.1104/pp.15.01237 PMC4936539

[tpj16380-bib-0048] Torres, M.A. , Dangl, J.L. & Jones, J.D.G. (2002) Arabidopsis gp91(phox) homologues *AtrbohD* and *AtrbohF* are required for accumulation of reactive oxygen intermediates in the plant defense response. Proceedings of the National Academy of Sciences of the United States of America, 99, 517–522. 11756663 10.1073/pnas.012452499 PMC117592

[tpj16380-bib-0049] Wu, F. , Chi, Y. , Jiang, Z. , Xu, Y. , Xie, L. , Huang, F. et al. (2020) Hydrogen peroxide sensor HPCA1 is an LRR receptor kinase in Arabidopsis. Nature, 578, 577–581. 32076270 10.1038/s41586-020-2032-3

[tpj16380-bib-0050] Zeng, W. & He, S.Y. (2010) A prominent role of the flagellin receptor FLAGELLIN‐SENSING2 in mediating stomatal response to *pseudomonas syringae* pv tomato DC3000 in Arabidopsis. Plant Physiology, 153, 1188–1198. 20457804 10.1104/pp.110.157016 PMC2899927

[tpj16380-bib-0051] Zhang, C. , Xie, Q. , Anderson, R.G. , Ng, G. , Seitz, N.C. , Peterson, T. et al. (2013) Crosstalk between the circadian clock and innate immunity in Arabidopsis. PLoS Pathogens, 9, e1003370. 23754942 10.1371/journal.ppat.1003370 PMC3675028

[tpj16380-bib-0052] Zhang, J. , Shao, F. , Li, Y. , Cui, H. , Chen, L. , Li, H. et al. (2007) A *pseudomonas syringae* effector inactivates MAPKs to suppress PAMP‐induced immunity in plants. Cell Host & Microbe, 1, 175–185. 18005697 10.1016/j.chom.2007.03.006

[tpj16380-bib-0053] Zhang, X. , Miao, Y.C. , An, G.Y. , Zhou, Y. , Shangguan, Z.P. , Gao, J.F. et al. (2001) K^+^ channels inhibited by hydrogen peroxide mediate abscisic acid signaling in Vicia guard cells. Cell Research, 11, 195–202. 11642404 10.1038/sj.cr.7290086

