# Supplementary material for: RBOHF activates stomatal immunity by modulating both reactive oxygen species and apoplastic pH dynamics in Arabidopsis
Source: Plant J. 2023 Jul 14;116(2):404–15. doi: 10.1111/tpj.16380 (PMC10952706; doi:10.1111/tpj.16380)
Supplement: Supplementary file 1 — Figure S1. Flg22‐triggered roGFP2‐Orp1 oxidation in pavement cells and guard cells. Figure S2. The NADPH oxidase RBOHF activates stomatal immunity. Figure S3. The rbohF mutant is partly defective in ABA‐ and H2O2‐mediated stomatal closure. Figure S4. Genotyping by PCR of rbohD and rbohF dSpm transposon mutants. Figure S5. Genotyping by PCR of rbohD and rbohF Salk T‐DNA mutants. [file TPJ-116-404-s001.pdf]

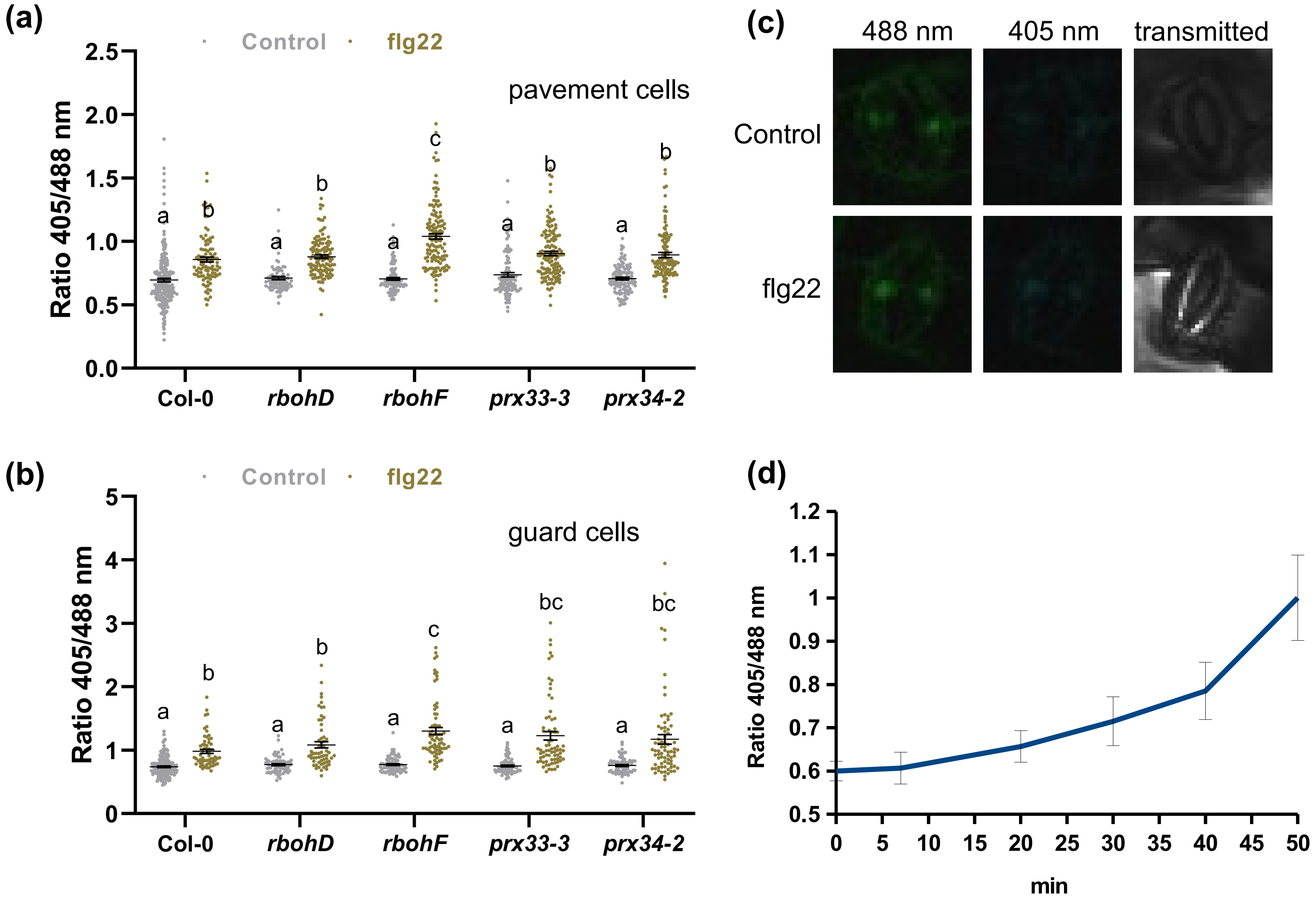

Figure S1. flg22-triggered roGFP2-Orp1 oxidation in pavement cells and guard cells.
(a-b), Oxidation state of roGFP2-Orp1 in Col-0 WT, *rbohD*, *rbohF*, *prx33-3* and *prx34-2* pavement cells (a) and guard cells (b) in response flg22. Leaf discs were exposed to control solution or 1 μM flg22 for 60 min and the ratio 405/488 nm of pavement cells or stomata selected as ROIs was quantified from images of the fluorescence emission at 517 ± 9 nm following excitation at 488 and 405 nm. Data are means ± SE (n ≥ 50) from a representative experiment. Different letters indicate significant differences at $P < 0.001$ (a) and $P < 0.05$ (b) based on a Tukey's HSD test.
(c) Representative images of the fluorescence emission at 517 ± 9 nm following excitation at 488 and 405 nm in selected Col-0 WT guard cells after treatment with Control solution or 1 μM flg22 for 1 h.
(d) Kinetics of roGFP2-Orp1 oxidation in WT guard cells in response to flg22. Leaf discs from Col-0 WT were exposed at t = 0 min to 1 μM flg22 and the ratio 405/488 nm of guard cells was measured over time by confocal microscopy. Data are means ± SE from a representative experiment (n = 7).

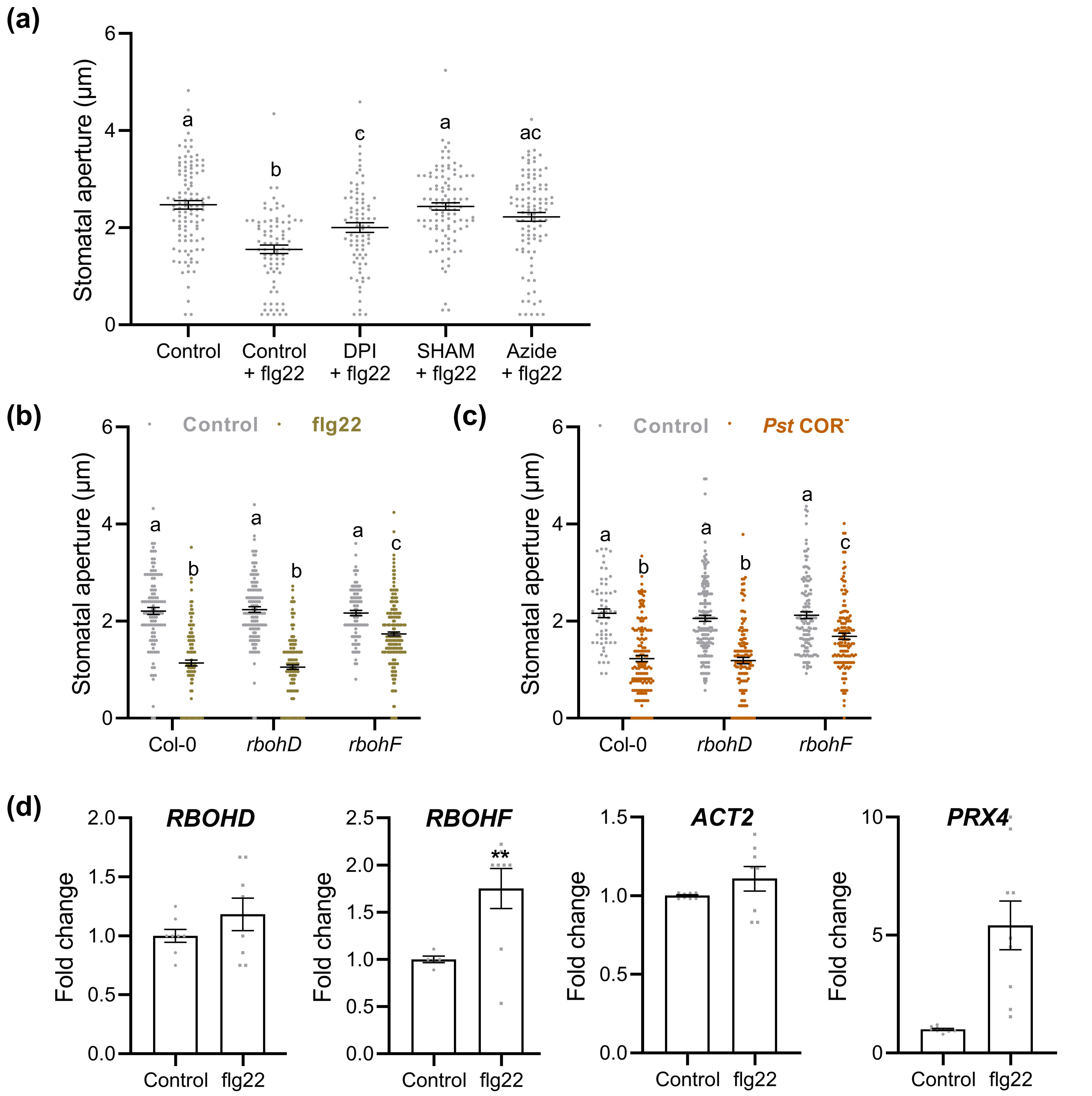

Figure S2. The NADPH oxidase RBOHF activates stomatal immunity.
(a) Stomatal aperture in epidermal peels of Col-0 WT pre-treated for 30 min with Control (0.1% DMSO), 20 µM DPI, 2 mM SHAM, or 1 µM sodium azide and exposed to Control solution or 5 µM flg22 for 2 h. Data are means ± SE (n ≥ 80) from a representative experiment.
(b) Stomatal apertures in WT Col-0, *rbohD*, and *rbohF* epidermal peels exposed to Control solution or 5 µM flg22 for 2 h.
(c) Stomatal apertures in Col-0 WT, *rbohD*, and *rbohF* epidermal peels exposed to Mock control (10 mM $MgCl_2$) or 10^8 cfu/ml COR-deficient *Pst* DC3000 (*Pst* COR^-) bacteria for 2 h. In (b) and (c) data are means ± SE (n ≥ 100) from a representative experiment. Different letters indicate significant differences at $P < 0.001$ (b-c) and $P < 0.05$ (a) based on a Tukey's HSD test.
(d) *RBOHF* expression is induced by flg22 in guard cells. Expression analysis of *RBOHD*, *RBOHF,* the reference gene *ACT2,* and the PTI marker gene *PRX4* by RT-qPCR in guard cell protoplasts isolated from leaves of 5-week-old Col-0 plants incubated in stomatal buffer for 2 h without (Control) or with 1 µM flg22. Transcript levels were normalized to *UBQ1*. The changes in transcript levels are relative to Control treatment (expression value = 1). Error bars indicate SE of six independent experiments (n = 6). Asterisks indicate statistically significant differences between Control and flg22 treatments based on a two-tailed Student's t-test (**$P < 0.01$).

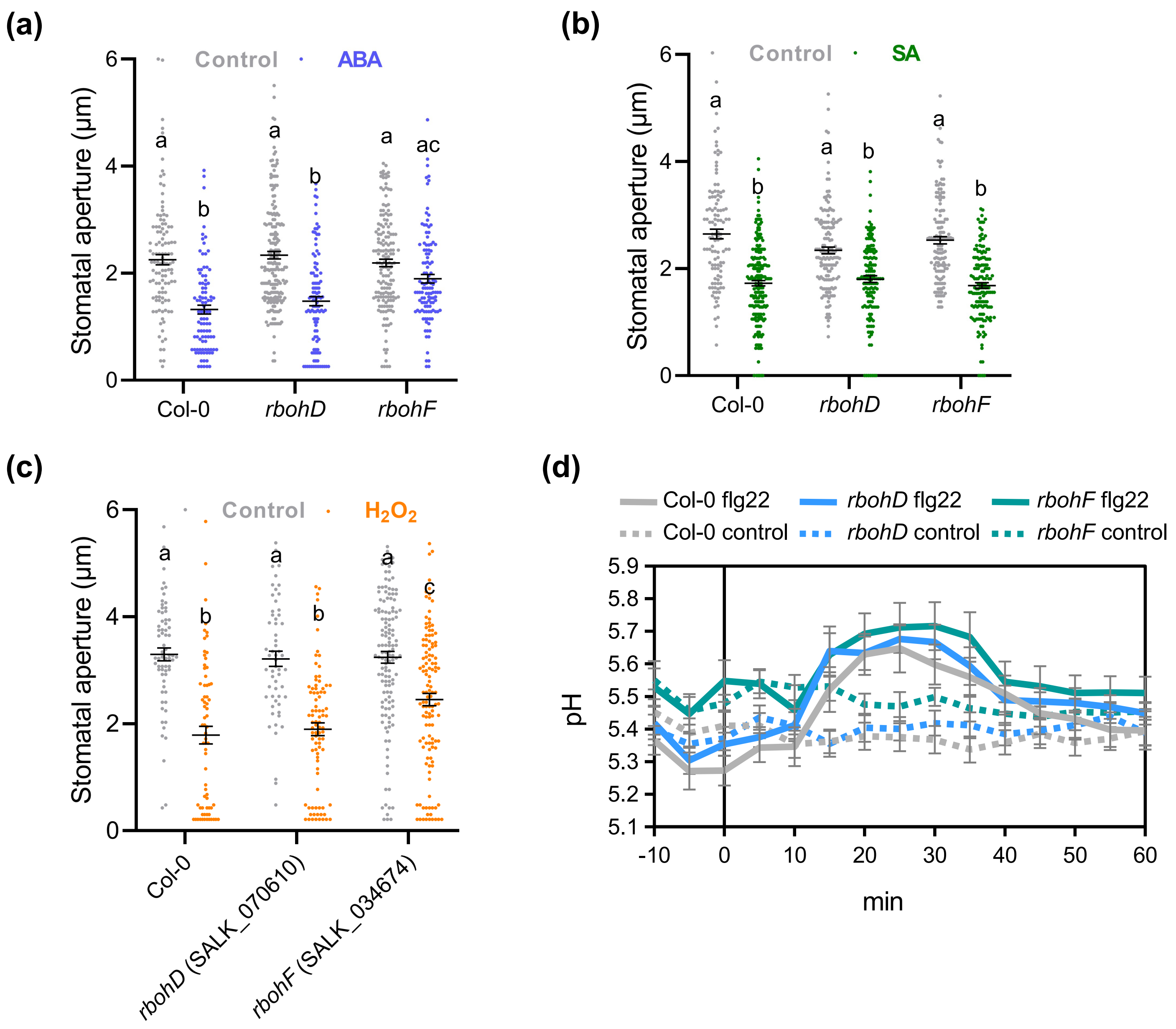

Figure S3. The *rbohF* mutant is partly defective in ABA- and H_2O_2-mediated stomatal closure.

(a) Stomatal apertures in Col-0 WT, *rbohD* and *rbohF* epidermal peels exposed to Control solution (0.01% ethanol) or 1 µM abscisic acid (ABA) for 2 h.

(b) Stomatal apertures in WT Col-0, *rbohD* and *rbohF* epidermal peels exposed to Control solution (0.01% ethanol) or 10 µM salicylic acid (SA) for 2 h. In (a) and (b) data are means ± SE (n ≥ 100) from a representative experiment and different letters indicate significant differences at $P < 0.01$ (a) and $P < 0.001$ (b) based on a Tukey's HSD test.

(c) Stomatal apertures in WT Col-0, *rbohD* (SALK_070610 allele) and *rbohF* (SALK_034674 allele) epidermal peels exposed to Control or 100 µM H_2O_2 for 2 h. Data are means ± SE (n ≥ 60) from a representative experiment. Different letters indicate significant differences at $P < 0.001$ based on a Tukey's HSD test.

(d) Kinetics of flg22-induced leaf apoplastic alkalinisation in Col-0 WT, *rbohD* and *rbohF* mutants. Oregon green dextran-infiltrated leaf discs were exposed at t = 0 min to control solution or 1 µM flg22, the apoplastic pH was measured over time by multiwell fluorimetry. Data are means ± SE (n ≥ 18) from 3 independent experiments. No significant differences between flg22-treated Col-0 WT, *rbohD* and *rbohF* was found between 10 and 60 min based on 2-way ANOVA and uncorrected Fisher's LSD analyses for each time point.

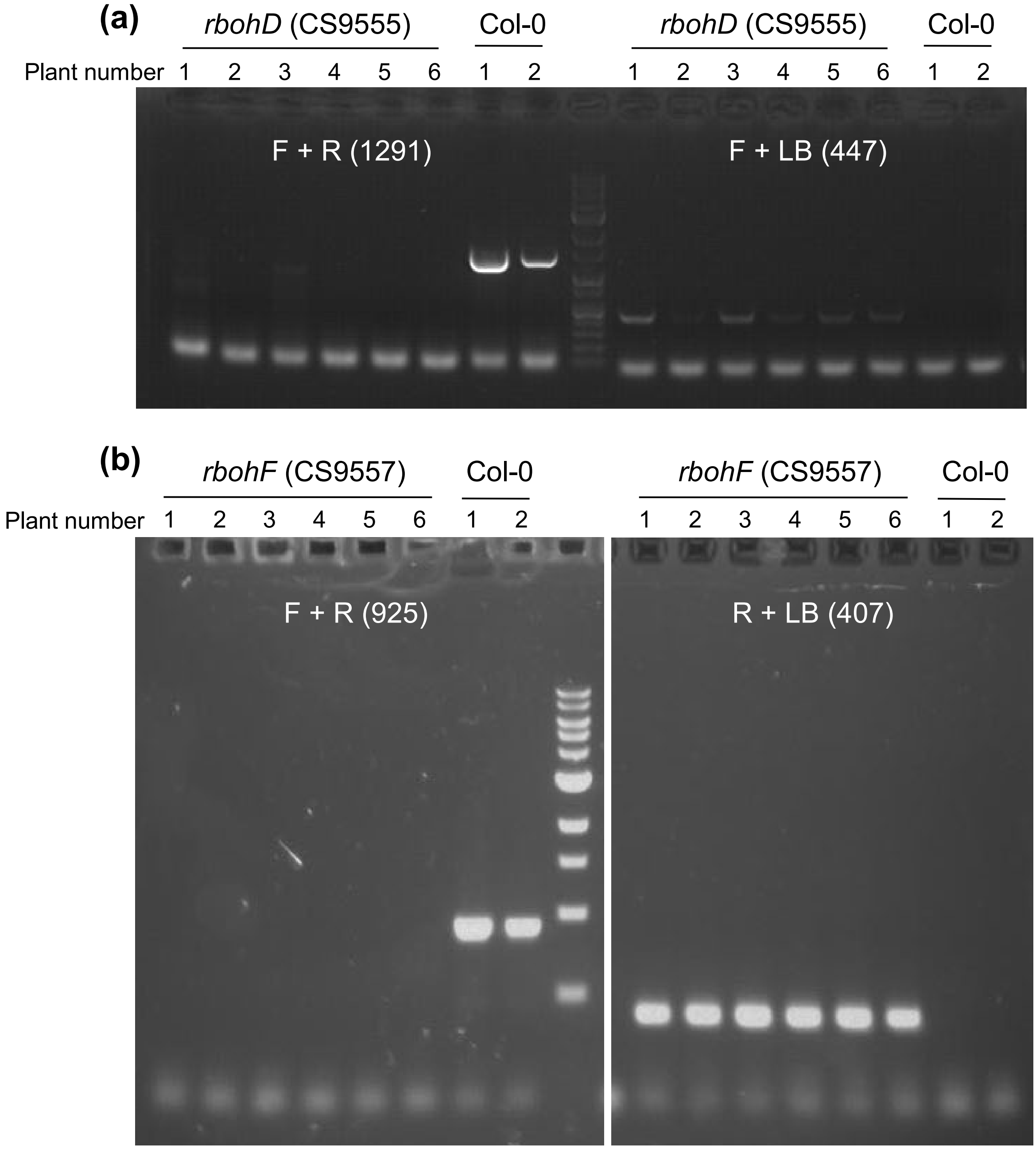

Figure S4. Genotyping by PCR of *rbohD* and *rbohF dSpm* transposon mutants.
(a-b) Representative images of PCR for genotyping the *rbohD* CS9555 (a) and *rbohF* CS9557 (b) mutants (Torres et al., 2002). Wild-type allele was amplified with gene specific forward and reverse primers (F + R), and T-DNA mutant allele was amplified with a gene specific primer (F or R) and the DsLox T-DNA left border (LB) primer. The expected size of amplicons is indicated under brackets. Col-0 wild-type genomic DNA was used as a control.

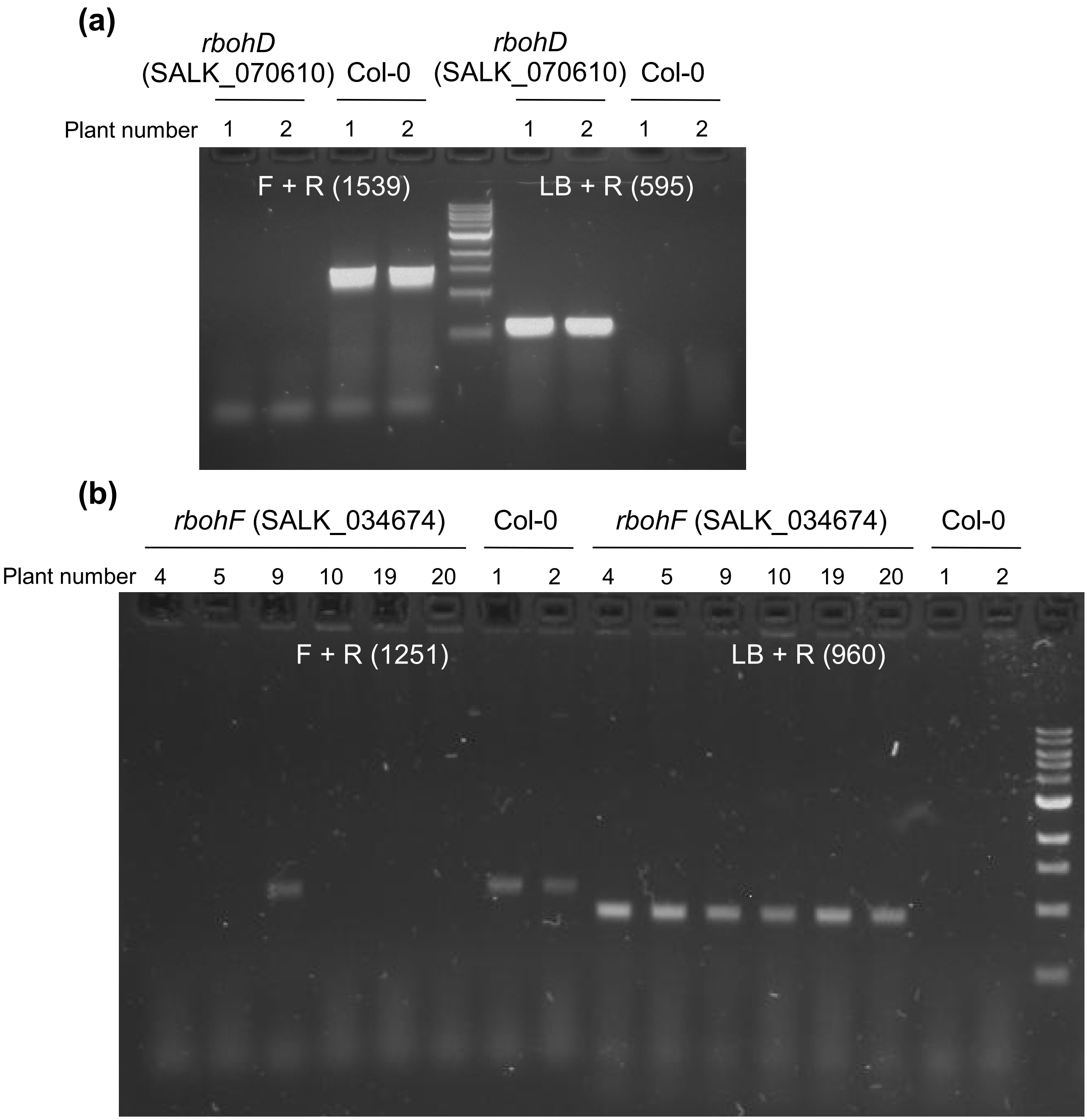

Figure S5. Genotyping by PCR of *rbohD* and *rbohF* Salk T-DNA mutants.

(a-b) Representative images of PCR for genotyping the *rbohD* SALK_070610 (a) and *rbohF* SALK_034674 (b) mutants. Wild-type allele was amplified with gene specific forward and reverse primers (F + R), and T-DNA mutant allele was amplified with a gene specific reverse primer (R) and the Salk T-DNA left border (LB) primer. The expected size of amplicons is indicated under brackets. Col-0 wild-type genomic DNA was used as a control.
