# Supplementary material for: RBOHF activates stomatal immunity by modulating both reactive oxygen species and apoplastic pH dynamics in Arabidopsis
Source: Plant J. 2023 Jul 14;116(2):404–15. doi: 10.1111/tpj.16380 (PMC10952706; doi:10.1111/tpj.16380)
Supplement: Supplementary file 3 — Table S2. PCR primers used for genotyping and RT‐qPCR. [file TPJ-116-404-s002.doc]

**Table S2.** PCR primers used for genotyping and RT-qPCR.

| **Name** | **Forward primers 5' to 3'** | **Reverse primers 5' to 3'** |
| --- | --- | --- |
| **Genotyping** |  |  |
| *rbohD* (CS9555) | CATGGGTTATTGCGTTTGTGTCGCCAA | GGATACTGATCATAGGCGTGGCTCCA |
| *rbohF* (CS9557) | ACTTCCGATATCCTTCAACCAACTCTTTG | CTCTCGTCGTTGATTTGTGACCAATACT |
| *rbohD* (SALK_070610) | GGCATCTGTGGTGGCCTCTTTAC | CCTCGTCTAACTCCGAAATCTCGT |
| *rbohF* (SALK_034674) | CATCTATCGCTCCGATTTCGCTC | AGCCAAGTCTTTCAGGATCTAACTC |
| *prx33-3* | TGGAAATGCAAATTCAGCCCGA | CAGATCGAAATCCACTAAGACG |
| *prx34-2* | CACCCCTACCTTCTACGATAG | CCATTTGTTCCTCTGAAGCAAG |
| Salk T-DNA | TGGTTCACGTAGTGGGCCATCG |  |
| GABI-Kat T-DNA | ATATTGACCATCATACTCATTGC |  |
| DsLox T-DNA | GCTCTTGCTAAGCTCCTCGAGTT |  |
| **RT-qPCR** |  |  |
| RBOHD | GACGATGAGTACGTGGAGATCA | GGAGGTGGTGTTGTTGAGGCT |
| RBOHF | CATCTATCGCTCCGATTTCGCT | CTCTCGTCGTTGATTTGTGACCA |
| PRX4 | GCGTTTAGGGCTATCGCAGAC | AAGCCTACCTTTGAACGTGAGG |
| ACTIN2 | GGTAACATTGTGCTCAGTGGTGG | AACGACCTTAATCTTCATGCTGC |
| UBQ1 | GCTTGCTCGTAAGTACAATCAGG | GGCCTCAACTGGTTGCTGTGA |
